# Age at menarche, age at menopause, reproductive years and risk of fatal stroke occurrence among Chinese women: the Guangzhou Biobank Cohort Study

**DOI:** 10.1186/s12905-021-01579-9

**Published:** 2021-12-28

**Authors:** Zhi-bing Hu, Ze-xiong Lu, Feng Zhu

**Affiliations:** 1 Department of Internal Medicine and Central Laboratory, Guangzhou Twelfth People’s Hospital, No. 1 Tianqiang St., Huangpu Rd., Guangzhou, 510620 China; 2 Department of Internal Medicine, Sanya Central Hospital, No. 1154 Jiefang Rd., Sanya, 572019 China

**Keywords:** Stroke, Ischaemic, Menarche, Menopause, Reproductive

## Abstract

**Background:**

The relationship between women’s reproductive characteristics and stroke events is unclear. We aimed to investigate age at menarche, age at menopause and number of reproductive years in relation to fatal stroke occurrence in the Guangzhou Biobank Cohort Study.

**Methods:**

In total, 16,504 postmenopausal women without stroke, heart disease or a cancer history at baseline were included and followed up for a median of 12.0 years. After review of available records, 222 stroke deaths were recorded. Cox proportional hazards regression was used to assess the associations between the risk of fatal stroke occurrence and age at menarche, age at menopause and number of reproductive years.

**Results:**

In the whole cohort, compared with those aged 15 years at menarche, an increased risk of fatal stroke among women at menarche showed respectively in those aged 12 years (aHR (adjusted hazard ratio) = 1.86, 95% confidence interval (CI) 0.96–3.60), aged 13 years (aHR = 1.69, 95% CI 0.98–2.92), aged 17 years (aHR = 1.83, 95% CI 1.10–3.05) and aged ≥ 18 years (aHR = 1.66, 95% CI 1.03–2.70), wherein the associations revealed an atypically U-shaped; similar U-shaped association to the cohort of postmenopausal women born before 1940 released a range of incremental risks of fatal stroke in women at menarche aged ≤ 12 years (aHR = 3.68, 95% CI 1.68–8.05), aged 13 years (aHR = 2.11, 95% CI 1.02–4.34), aged 14 years (aHR = 2.07, 95% CI 1.04), aged 17 years (aHR = 2.30, 95% CI 1.20–4.39) and aged 18 years (aHR = 2.50, 95% CI 1.37–4.57), respectively. Compared with menopausal women aged 51–52 years, those aged < 43 years at menopause had an increased risk for fatal stroke among postmenopausal women born in and after 1940 (aHR = 1.64, 95% CI 0.97–2.78) and postmenopausal women born before 1940 (aHR = 1.97, 95% CI 1.05–3.69). Additionally, compared with those with 32–34 reproductive years, women with ≤ 28 reproductive years had an increased risk for fatal stroke in the whole cohort (aHR = 1.91, 95% CI 1.28–2.86) and the cohort of postmenopausal women born before 1940 (aHR = 1.79, 95% CI 1.15–2.80).

**Conclusions:**

Younger and older age at menarche, younger age at menopause and fewer reproductive ages were related to an increased risk of fatal stroke in postmenopausal women.

**Supplementary Information:**

The online version contains supplementary material available at 10.1186/s12905-021-01579-9.

## Background

In China, the life expectancy of women is 79.9 years, which is approximately 6.5 years longer than that of men [1]. Stroke is a leading cause of death in China [2] and is considered to be a sexually dimorphic disease [3]. Compared with men, women more easily suffer from cerebrovascular disease [4], have a higher stroke incidence [5] and have poorer functional outcomes for intravenous thrombolysis (IVT)-treated ischaemic stroke [6]. One study revealed that sex had a modifying effect on mortality in diabetic patients with first-ever ischaemic stroke, showing an increased risk of long-term mortality among women [7].

Menarche is the beginning, and menopause is the end of a woman’s reproductive timeline, and the reproductive years, which is the interval between menarche and menopause, is a woman’s natural reproductive window. Recent studies have reported that early menarche is associated with an increased mortality or incidence risk of stroke [8–15], though there are conflicting reports [12, 16–23]. Additionally, a U-shaped association [24] and an increased risk of stroke incidence have been found in those with late menarche [19, 25]. However, early menarche showed a 16% lower but an 18% higher risk of stroke among women born during the 1920s–1940s and women born in the 1950s, respectively, and a U-shaped association among women born in the 1960s-1970s [14]. Fewer reproductive years was also associated with stroke mortality or incidence [10, 12, 25–27], though there were inconsistencies in results [8, 19]. Thus, these previous studies on women showed a mixed relationship between reproductive characteristics and stroke events.

Besides an anomalous increase during the 1958–61 national famine, age at menarch droped but age at menopause increased with years among Chinese women [28]. Although one study in age at menarch was released [14], the relationship between Chinese women’s reproductive characteristics and stroke events is not comprehensive. Here, we aimed to systematically assess the associations between age at menarche, age at menopause, and number of reproductive years and the risk of fatal stroke occurrence in healthy postmenopausal Chinese women.

## Methods

### Data source and participants

Details of the Guangzhou Biobank Cohort Study (GBCS) have been reported previously [29]. Briefly, this is an on-going prospective cohort study including permanent Guangzhou residents aged 50 years or older that aims to examine environmental and genetic determinants of chronic diseases. The GBCS is a collaborative project among the Guangzhou 12th Hospital and the Universities of Hong Kong and Birmingham. Those who were receiving treatment for life-threatening diseases, such as cancer, or did not provide informed consent, were excluded. A total of 30,518 older Chinese individuals including 22,067 women in Guangzhou were recruited at baseline. The baseline (from September 1st, 2003, to February 28th, 2008) included a face-to-face computer-assisted interview by trained nurses on lifestyle, family and personal medical history; assessments of anthropometrics and blood pressure; and a series of laboratory tests, including fasting plasma glucose and lipids. Physical activity was assessed by means of the Chinese version of the International Physical Activity Questionnaire (IPAQ) and was classified into inactive, moderately active and physically active [30].

### Exposure indicators

The ages at menarche and menopause, which is the end of a woman's menstrual cycle and fertility wherein both natural and induced menopause included by the removal of uterus and ovaries, were recorded in years (per the Gregorian calendar and related interpretation of age) and rounded during data collection to the nearest year (e.g., 13 years represents the onset of menarche from 12 years 6 months to 13 years 5 months) [31]. The number of reproductive years was generated by subtracting age at menarche from age at menopause. Data on each woman’s history of oral contraceptive (OC) use (ever, never), hormone replacement therapy, hysterectomy and ovarian or breast surgery were also recorded.

### Outcome indicators

Information on underlying causes of death up to December 31st, 2017, was obtained mostly via record linkage with the Guangzhou Centres for Disease Control and Prevention (GZCDC). Because there was no other information for stroke severity, infarct volume, lesion site or infectious complications, fatal stroke occurrence was chosen as the primary outcome of this study. Death causes were coded according to the 10th revision of the International Classification of Diseases (ICD) as follows: I60 ~ I69 for stroke; I60.0 ~ I62.9 and I69.0 ~ I69.2 for haemorrhagic stroke; I63.0 ~ I63.9 and I69.3 for ischaemic stroke; and the other codes for unclassified stroke. When the death certificates were not issued by medical institutions, the causes were verified by GZCDC as part of their quality assurance programmed by cross-checking past medical history and conducting verbal autopsy by 5 senior clinicians from Guangzhou Twelfth People’s Hospital, the Universities of Hong Kong, China, and Birmingham, UK.

To examine the extent to which baseline factors explained the associations of stroke, ischaemic stroke and haemorrhagic stroke, we included the factors in different models. Model 1 was a crude hazard ratio model without adjustment for any confounders. Model 2 contained a multivariate adjustment for factors including age, diabetes, hypertension, dyslipidaemia, smoking, alcohol consumption, physical activity, body mass index, self-rated health, education, job, family income, number of children and oral contraceptive pill use.

### Statistical analysis

Cox proportional hazards regression was used to analyse the association between age at menarche and age at menopause, reproductive years and the risk of fatal stroke. The age at menarche was categorized into 7 age groups: ≤ 12, 13, 14, 15, 16, 17, and ≥ 18 years [14]. The age at menopause was categorized into 5 age groups: < 43, 43–47, 48–50, 51–52, and ≥ 53 years [26]. The reproductive years were categorized into 5 age groups: ≤ 28, 29–31, 32–34, 35–37, and ≥ 38 years. Fewer reproductive years was defined as the lowest deciles [19]. We chose 1940 as cut-point and added two birth cohorts(details on the two birth cohorts showed on Additional file 1: Table 4), because age at menarche among Chinese women droped with years, besides an anomalous increase during the 1958–61 national famine, and the menarche ended among most women born before 1940 [14]. Covariates included age, diabetes, hypertension, dyslipidaemia, smoking, alcohol consumption, physical activity, body mass index, self-rated health, education, job, family income, number of children and oral contraceptive pill use. Similar to a previous Chinese population study [28], our cohort showed that age at menarche decreased and that age at menopause increased in younger generations. Thus, further analysis was conducted by birth cohort; by smoking status (never smoker with additional adjustments for childhood passive smoking exposure, which is linked to an earlier age at both menarche [32] and menopause [33], and stroke mortality [34]). A sensitivity analysis was conducted after exclusion of those with removal of the uterus, ovaries, or breast masses (benign or malignant) or those who received hormone replacement therapy. All analyses were performed using STATA (version 14.0; StataCorp LP, College Station, TX, USA). All p values were 2 sides, and statistical significance was defined as *P* < 0.05.

## Results

### Baseline characteristics

**Fig. 1:**
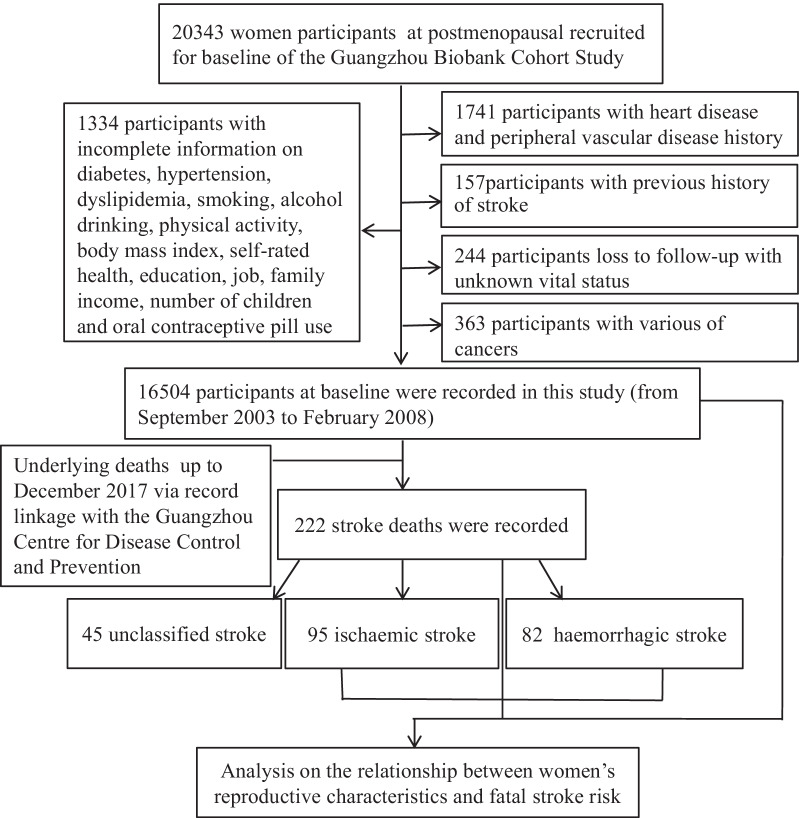
Flow diagram of participants selected for the analysis of this study

In total, 22,067 (72.5% of whole participants, original sample size) female participants comprising 20,343 (sample size without other exclusion criteria) postmenopausal women were screened. Among 2419 patient exclusions, 244 were lost to follow-up and had an unknown vital status, 157 had a history of stroke, 1741 had heart disease or peripheral vascular disease, 363 had cancer, and 1334 had incomplete information on diabetes, hypertension, dyslipidaemia, smoking, alcohol consumption, physical activity, body mass index, self-rated health, education, job, family income, number of children and oral contraceptive pill use. A total of 16,504 participants who were free of stroke at baseline were included in this study, they had higher rates of ≤ primary education and manual jobs, lower rates of hypertension, and had a lower family income, compared with those exclusions (Additional file 1: Table 5). After a median follow-up of 12.0 (Q25 (the 25th quartile):10.7, Q75 (the 75th quartile):13.4) years, 222 stroke deaths (95 ischaemic, 82 haemorrhagic and 45 unclassified) were recorded (Fig. 1). Among the postmenopausal women, the mean age was 61.4 (6.8) years, and the age at menarche was 15.1 (2.1) years, with 15.7 (2.1) years among those born before 1940 and 14.9 (2.0) years among those born in 1940 or later); the age at menopause was 49.5 (3.8) years, with 48.9 (4.1) years among those born before 1940 and 49.7 (3.6) years among those born in 1940 or later); and the number of reproductive years was 34.3 (4.3) years, with 33.2 (4.6) years among those born before 1940 and 34.8 (4.0) years among those born in 1940 or later (Additional file 1: Tables 4–5).

The baseline characteristics of the participants according to age at menarche are presented in Table 1. Compared with those aged 15 years at menarche, those with early menarche (≤ 12) were younger; had lower rates of ≤ primary education and manual jobs; had a higher family income, lower proportion of women with a number of children born > 3, and a higher number of reproductive years; and had higher proportions of current smokers, alcohol consumers and women with physical activity. Compared with those aged 15 years at menarche, those with late menarche (≥ 18) were older, comprised more current smokers, and were more likely to engage in physical activity; had higher rates of ≤ primary education and manual jobs; had a higher proportion of women with a number of children > 3 and lower family income; had lower rates of hypertension, diabetes, current drinking, and oral contraceptive pill use; and had fewer reproductive years.

**Table 1:** Baseline characteristics by age at menarche of participants in the GBCS

Characteristics	Overall	Age at menarche (years)						
		≤ 12	13	14	15	16	17	≥ 18
Number, n	16,504	1368	2662	2849	2787	2667	1720	2451
Age (years)^b^	61.4	59.0	59.4	60.2	61.7	62.1	63.1	64.1
Hypertension, %^b^	27.0	27.6	27.8	27.9	27.6	27.6	26.7	23.8
Diabetes, %^a^	13.1	13.7	12.3	13.3	13.6	13.8	14.5	11.3
Dyslipidaemia, %^b^	85.7	87.4	87.6	86.8	85.9	85.2	84.2	82.7
BMI (kg/m^2^)^b^	23.8	24.3	23.9	23.8	23.8	23.8	23.8	23.6
Good/very good self-rated health, %^c^	82.7	82.6	83.5	83.0	82.5	82.5	81.2	82.8
Oral contraceptive pill use (ever), %^b^	17.8	17.6	18.9	19.7	17.4	19.0	16.7	14.2
Current smoker, %^b^	1.9	2.0	1.7	1.5	1.7	2.5	1.6	2.3
Current drinking, %^b^	20.6	26.4	23.0	22.2	19.9	18.6	18.1	17.5
Active physical activity, %^c^	52.6	52.1	53.0	52.2	50.9	52.2	54.1	54.1
Education ≤ primary, %^b^	49.9	31.5	33.3	38.6	50.0	58.2	65.1	71.8
Manual job, %^b^	54.5	43.3	47.1	47.7	54.8	58.3	62.2	66.6
Family income < 30,000 CNY/year, %^b^	38.3	33.5	34.9	35.0	38.0	41.6	43.7	41.3
Number of children born > 3, %^b^	21.4	8.6	10.9	13.2	21.2	26.5	31.8	36.7
Age at menopause (years)^b^	49.5	49.0	49.4	49.6	49.6	49.6	49.5	49.5
Reproductive years (years)^b^	34.3	37.2	36.4	35.6	34.6	33.6	32.5	30.9

^a^*P* < 0.05; ^b^*P* < 0.01; ^c^*P* > 0.05; hypertension: systolic blood pressure, ≥ 140 mmHg, diastolic blood pressure, ≤ 90 mmHg, medication or diagnosed; diabetes: fasting blood glucose ≥ 7 or medication or diagnosis; dyslipidaemia: total cholesterol ≥ 5.2 mmol/L, triglyceride ≥ 1.7 mmol/L, low density lipoprotein ≥ 3.4 mmol/L, high density lipoprotein < 1.0 mmol/L, medication or diagnosis; *BMI* body mass index

### Menarche in relation to fatal stroke occurrence

Menarche in relation to fatal stroke occurrence is presented in Table 2, which seemed to be U-shaped. In the whole cohort, compared with those aged 15 years at menarche, an increased risk of fatal stroke among women at menarche showed respectively in those aged 12 years (aHR = 1.86,95% CI 0.96–3.60), aged 13 years (aHR = 1.69, 95CI% 0.98–2.92), aged 17 years (aHR = 1.83, 95% CI 1.10–3.05) and aged ≥ 18 years (aHR = 1.66, 95% CI 1.03–2.70); similar U-shaped association to postmenopausal women born before 1940 released a range of incremental risks of fatal stroke in those at menarche aged ≤ 12 years(aHR = 3.68, 95% CI 1.68–8.05), aged 13 years (aHR = 2.11, 95% CI 1.02–4.34), aged 14 years (aHR = 2.07, 95% CI 1.04), aged 17 years (aHR = 2.30, 95% CI 1.20–4.39) and aged 18 years (aHR = 2.50, 95% CI 1.37–4.57). However, there were no significant associations between fatal stroke and menarche in those born in 1940 or later. All these associations were still observed in a sensitivity analysis after exclusions of patients with removal of the uterus, ovaries, or breast masses (benign or malignant) or who received hormone replacement therapy (Additional file 1: Table 1).

**Table 2:** Association between fatal stroke occurrence and age at menarche in the GBCS

	Age at menarche (years)						
	≤ 12	13	14	15	16	17	≥ 18
**Total cohort**	1368	2662	2849	2787	2667	1720	2451
No. of deaths	14 (0.010)	28 (0.011)	28 (0.010)	25 (0.009)	39 (0.015)	37 (0.022)	51 (0.021)
Model 1 (HR, 95% CI)	1.22 (0.64–2.35), *P* = 0.55	1.19 (0.70–2.05), *P* = 0.52	1.13 (0.66–1.94), *P* = 0.66	1.00	1.63 (0.99–2.69), *P* = 0.06	2.39 (1.44–3.97), *P* = 0.001	2.34 (1.45–3.77), *P* = 0.001
Model 2 (HR, 95% CI)	1.86 (0.96–3.60), *P* = 0.07	1.69 (0.98–2.92), *P* = 0.06	1.41 (0.82–2.42), *P* = 0.22	1.00	1.41 (0.85–2.34), *P* = 0.18	1.83 (1.10–3.05), *P* = 0.02	1.66 (1.03–2.70), *P* = 0.04
**Born < 1940**^**$**^** cohort**	261	511	669	898	887	677	1035
No. of deaths	12 (0.046)	16 (0.031)	20 (0.030)	14 (0.016)	27 (0.030)	28 (0.042)	47 (0.045)
Model 1 (HR, 95% CI)	3.01 (1.39–6.50), *P* = 0.005	1.96 (0.96–4.03), *P* = 0.07	1.90 (0.96–3.77), *P* = 0.07	1.00	1.92 (1.01–3.66), *P* = 0.05	2.63 (1.39–5.00), *P* = 0.003	3.04 (1.67–5.51), *P* < 0.001
Model 2 (HR, 95% CI)	3.68 (1.68–8.05), *P* = 0.001	2.11 (1.02–4.34), *P* = 0.04	2.07 (1.04–4.11), *P* = 0.04	1.00	1.69 (0.89–3.24), *P* = 0.11	2.30 (1.20–4.39), *P* = 0.01	2.50 (1.37–4.57), *P* = 0.003
**Born ≥ 1940**^**&**^** cohort**	1107	2151	2180	1889	1780	1043	1416
No. of deaths	2 (0.002)	12 (0.006)	8 (0.004)	11 (0.006)	12 (0.007)	9 (0.009)	4 (0.003)
Model 1 (HR, 95% CI)	0.33 (0.07–1.50), *P* = 0.15	0.97 (0.43–2.21), *P* = 0.95	0.65 (0.26–1.63), *P* = 0.36	1.00	1.17 (0.52–2.65), *P* = 0.71	1.49 (0.62–3.59), *P* = 0.38	0.48 (0.15–1.51), *P* = 0.21
Model 2 (HR, 95% CI)	0.37 (0.08–1.69), *P* = 0.20	1.14 (0.50–2.60), *P* = 0.76	0.70 (0.28–1.76), *P* = 0.45	1.00	1.14 (0.50–2.61), *P* = 0.75	1.35 (0.56–3.30), *P* = 0.51	0.42 (0.13–1.34), *P* = 0.14

^$^Postmenopausal women born before 1940 were analysed; ^&^Postmenopausal women born in 1940 or later were analysed; model 1: a crude hazard ratio model without adjustments; model 2: a multivariate adjusted model including age, diabetes, hypertension, dyslipidaemia, smoking, alcohol drinking, physical activity, body mass index, self-rated health, education, job, family income, number of children and oral contraceptive pill use

### Menopause in relation to fatal stroke occurrence

Table 3 shows menopause in relation to fatal stroke occurrence. Compared with menopausal women aged 51–52 years, those with earlier menopause at age < 43 years had an increased risk of fatal stroke (aHR = 1.64, 95% CI 0.97–2.78). Compared with menopausal women aged 51–52 years, postmenopausal women born before 1940 showed a significant association with the risk of fatal stroke for those of age < 43 (aHR = 1.97, 95% CI 1.05–3.69). However, there were no significant associations between fatal stroke and menopause in those born in 1940 or later. These associations were still observed in a sensitivity analysis after excluding uterus removal, ovaries, breast masses (benign or malignant) or hormone replacement therapy (Additional file 1: Table 2).

**Table 3:** Association between fatal stroke occurrence and age at menopause in the GBCS

	Age at menopause (years)				
	< 43	43–47	48–50	51–52	≥ 53
**Total cohort**	940	2805	6600	2928	3231
No. of deaths	26 (0.028)	42 (0.015)	88 (0.013)	31 (0.010)	35 (0.010)
Model 1 (HR, 95% CI)	2.58 (1.53–4.35), *P* < 0.001	1.40 (0.88–2.23), *P* = 0.16	1.25 (0.83–1.88), *P* = 0.29	1.00	0.98 (0.61–1.59), *P* = 0.94
Model 2 (HR, 95% CI)	1.64 (0.97–2.78), *P* = 0.06	1.09 (0.69–1.74), *P* = 0.71	1.10 (0.73–1.65), *P* = 0.66	1.00	0.99 (0.61–1.61), *P* = 0.96
**Born < 1940**^**$**^** cohort**	413	981	1996	752	796
No. of deaths	22 (0.053)	36 (0.037)	62 (0.031)	18 (0.024)	26 (0.033)
Model 1 (HR, 95% CI)	2.30 (1.23–4.28), *P* = 0.009	1.52 (0.86–2.67), *P* = 0.15	1.29 (0.77–2.18), *P* = 0.34	1.00	1.33 (0.73–2.42), *P* = 0.35
Model 2 (HR, 95% CI)	1.97 (1.05–3.69), *P* = 0.03	1.43 (0.81–2.52), *P* = 0.22	1.25 (0.74–2.11), *P* = 0.41	1.00	1.35 (0.74–2.47), *P* = 0.33
**Born ≥ 1940**^**&**^** cohort**	527	1824	4604	2176	2435
No. of deaths	4 (0.008)	6 (0.003)	26 (0.006)	13 (0.006)	9 (0.004)
Model 1 (HR, 95% CI)	1.24 (0.41–3.81), *P* = 0.71	0.55 (0.21–1.46), *P* = 0.23	0.94 (0.48–1.83), *P* = 0.86	1.00	0.59 (0.25–1.38), *P* = 0.22
Model 2 (HR, 95% CI)	1.00 (0.32–3.08), *P* = 0.99	0.52 (0.20–1.37), *P* = 0.19	0.92 (0.47–1.79), *P* = 0.80	1.00	0.54 (0.23–1.27), *P* = 0.16

^$^Postmenopausal women born before 1940 were analysed; ^&^Postmenopausal women born in 1940 or later were analysed; model 1: a crude hazard ratio model without adjustments; model 2: a multivariate adjusted model including age, diabetes, hypertension, dyslipidaemia, smoking, alcohol drinking, physical activity, body mass index, self-rated health, education, job, family income, number of children and oral contraceptive pill use

### Reproductive years in relation to fatal stroke occurrence

Table 4 shows reproductive years in relation to fatal stroke occurrence. Compared with those with a number of reproductive years from 32 to 34, those with a number of reproductive years ≤ 28 had an increased risk of fatal stroke (aHR = 1.91, 95% CI 1.28–2.86) in the whole cohort, and similar associations were found for fatal stroke (aHR = 1.79, 95% CI 1.15–2.80) in the cohort of postmenopausal women born before 1940. Moreover, no reproductive years in relation to fatal stroke were observed in the cohort born in 1940 or later. Such associations were still observed in a sensitivity analysis after exclusion of those who had removal of the uterus, ovaries, or breast masses (benign or malignant) or received hormone replacement therapy (Additional file 1: Table 3).

**Table 4:** Association between fatal stroke occurrence and reproductive years in the GBCS

	Duration of reproductive years				
	≤ 28	29–31	32–34	35–37	≥ 38
**Total cohort**	1511	2193	4025	5114	3661
No. of deaths	49 (0.032)	32 (0.015)	49 (0.012)	58 (0.011)	36 (0.010)
Model 1 (HR, 95% CI)	2.69 (1.81–4.00), *P* < 0.001	1.18 (0.76–1.85), *P* = 0.46	1.00	0.90 (0.61–1.33), *P* = 0.60	0.81 (0.52–1.24), *P* = 0.33
Model 2 (HR, 95% CI)	1.91 (1.28–2.86), *P* = 0.001	0.97 (0.62–1.52), *P* = 0.90	1.00	1.09 (0.74–1.61), *P* = 0.65	1.11 (0.72–1.72), *P* = 0.64
**Born < 1940**^**$**^** cohort**	713	878	1283	1293	771
No. of deaths	41 (0.058)	29 (0.033)	38 (0.030)	33 (0.026)	23 (0.030)
Model 1 (HR, 95% CI)	2.03 (1.30–3.15), *P* = 0.002	1.13 (0.70–1.83), *P* = 0.62	1.00	0.86 (0.54–1.36), *P* = 0.51	1.00 (0.60–1.68), *P* = 0.99
Model 2 (HR, 95% CI)	1.79 (1.15–2.80), *P* = 0.01	1.05 (0.65–1.71), *P* = 0.83	1.00	0.89 (0.56–1.42), *P* = 0.62	1.14 (0.67–1.93), *P* = 0.62
**Born ≥ 1940**^**&**^** cohort**	798	1315	2742	3821	2890
No. of deaths	8 (0.010)	3 (0.002)	11 (0.004)	25 (0.006)	13 (0.004)
Model 1 (HR, 95% CI)	2.51 (1.01–6.25), *P* = 0.047	0.56 (0.16–2.01), *P* = 0.37	1.00	1.50 (0.73–3.07), *P* = 0.27	1.10 (0.50–2.47), *P* = 0.81
Model 2 (HR, 95% CI)	2.11 (0.84–5.27)*, P* = 0.11	0.52 (0.14–1.86), *P* = 0.31	1.00	1.63 (0.79–3.36), *P* = 0.19	1.13 (0.51–2.55), *P* = 0.76

^$^Postmenopausal women born before 1940 were analyzed; ^&^Postmenopausal women born in 1940 or later were analyzed; model 1: a crude hazard ratio model without adjustments; model 2: a multivariate adjust model including age, diabetes, hypertension, dyslipidemia, smoking, alcohol drinking, physical activity, body mass index, self-rated health, education, job, family income, number of children and oral contraceptive pill use

## Discussion

In this study, we found that younger and older menarche, early menopause and fewer reproductive years were related to an increased risk of fatal stroke in Chinese postmenopausal women. The association was independent of age, diabetes, hypertension, dyslipidaemia, smoking, alcohol consumption, physical activity, body mass index, self-rated health, education, job, family income, number of children and oral contraceptive pill use.

Previous studies are inconsistent in terms of the association between age at menarche and stroke [8–12, 16–19, 24, 25]. In a previous cohort study of 37,965 Japanese postmenopausal women, age ≥ 17 years at menarche was associated with a increased risk of mortality from stroke compared with age at menarche ≤ 13 years [19], and a cohort study of 66,104 Korean women showed that women with age at menarche ≥ 17 years had higher risks for total stroke and thrombotic stroke compared with age at menarch of 13–14 years [25]. Our findings were similar in that late menarche was associated with an increased risk of fatal stroke occurrence. A U-shaped relationship between both early and late menarche and the increased risk of stroke incidence was observed in 1.2 million British women aged 50 to 64 years without prior heart disease, stroke, or cancer. This finding is the same as ours, wherein fatal stroke occurrence occurred in older women born before 1940 but not born after the 1960s in China [14]. However, our results were not conclusive due to very small number of events, and large prospective studies are needed to replicate our findings.

It remains unclear why menarche is related to stroke events. We observed that menarche in relation to fatal stroke occurrence was independent of removal of the uterus, ovaries, or breast masses (benign or malignant) or use of hormone replacement therapy, and.the associations remained after further analysis of never smoking and additional adjustments for childhood passive smoking exposure, which suggests that these associations may be influenced by other risk factors. As in a previous study addressing early menarche in relation to metabolic syndrome and its components [31, 35], our results were independent of BMI, hypertension, diabetes, and other lifestyle and cardiovascular risk factors. Moreover, other unmeasured factors, including lower birth weight, higher body weight and weight gain in infancy and childhood, may be related to age at menarche and increase the risk of early menarche [36]. Additionally, in utero exposure and active physical activity during pregnancy [37] may be related to a modest delay in age at menarche among offspring. Anorexia, malnutrition and premenarchal intense exercise training are related to delayed menarche [38, 39]. Thus, a U-shaped association here may be due to unmeasured but unrelated genetic factors [40].

There is an inconsistent association between menopause and stroke [10–13, 15, 19–23, 26]. Women aged 80 years or older, but not those aged < 80 years, who were < 40 years old at menopause had a 2.29 times higher stroke mortality risk than those aged 45–49 years at menopause [13]. Here, a lower age at menopause (< 43 years) was associated with higher fatal stroke occurrence in older generations born before 1940 but not in those born in 1940 or later. However, other reports show inconsistent findings [19–21, 23].

Early menopause in relation to the risk of fatal stroke occurrence is due mainly to changes in endogenous steroid hormones, especially oestradiol, after menopause [41, 42]. Oestradiol should be a protective factor in stroke, although hormone replacement therapy does not play a beneficiary role [43, 44]. In our study, the proportion of induced menopause is 8.6%, and a similar association released regardless of whether a series of factors or adjusted for lifestyle and cardiovascular risk factors were excluded. For aught we know, the effect of early menopause on fatal stroke may due to other risk factors.

The reproductive years constitute the interval between menarche and menopause. Most studies have reported that a low number of reproductive years is related to an increased fatal stroke or stroke incidence [10, 12, 25–27], though there are contradictory reports [8, 19]. Here, fewer reproductive years (≤ 28 years) was associated with an increased fatal stroke occurrence, which is consistent with a Korean cohort study addressing an increased risk for developing stroke and thrombotic stroke [25] and a case–control study on nonembolic ischaemic stroke risk [12]. Similar associations for fatal stroke were reported only by three teams: one in China and two in the United States [10, 26, 27]. An inconsistent relationship between reproductive years and fatal stroke occurrence was observed in China and Japan [8, 19].

In this study, 1940 was chose as a cut-point and two birth cohorts were added (the cohort born ≥ 1940, the cohort born < 1940) for further analysis (Additional file 1: Table 4). The reasons are that age at menarche among Chinese women droped with years, besides an anomalous increase during the 1958–61 national famine, and the menarche ended among most women born before 1940 [14]. Corresponding results showed that the cohort born < 1940 had more fatal stroke occurrence than the cohort born ≥ 1940 (Additional file 1: Table 4). This revealed that the younger cohort was not old enough to develop stroke events, although a limitation of low statistical power was released.

In our study, a median follow-up of 12.0 years makes this a large, prospective design for a study of the general population in South China, and individuals completed a physical examination and questionnaire involving a total of 800 questions. Thus, the acquired information allows for systemic adjustments for additional potential confounding factors. However, there are limitations in this study. First, we obtained only the death information via record linkage with the GZCDC. The results of our study with death as the only outcome are obviously weakened because of the lack of analysis of other clinical outcomes of stroke events. Second, among a series of potential confounders, there may be some inaccuracies in the risk factors, such as self-reported age at menarche and menopause, from the multi-year recall [45–47]. Third, the subjects could not represent Chinese individuals due to the limitations involving the general population in South China in this study. Finally, the small number of deaths limited the strength of this study to address fatal stroke, especially in a younger cohort.

## Conclusion

Age at menarche, age at menopause and number of reproductive years were related to the risk of fatal stroke occurrence. The burden of stroke may be increased in certain populations, and such women should be given more attention during the decade after menopause.

## Supplementary Information

**Additional file 1.** **Table 1.** Association between fatal stroke occurrence and age at menarche after a series of exclusions in the GBCS$: Postmenopausal women born before 1940 were analyzed; model 1: a crude hazard ratio model without adjustments; model 2: a multivariate adjust model including age, diabetes, hypertension, dyslipidemia, smoking, alcohol drinking, physical activity, body mass index, self-rated health, education, job, family income, number of children and oral contraceptive pill use. **Table 2.** Association between fatal stroke occurrence and age at menopause after a series of exclusions in the GBCS. $: Postmenopausal women born before 1940 were analyzed; model 1: a crude hazard ratio model without adjustments; model 2: a multivariate adjust model including age, diabetes, hypertension, dyslipidemia, smoking, alcohol drinking, physical activity, body mass index, self-rated health, education, job, family income, number of children and oral contraceptive pill use. **Table 3.** Association between stroke mortality and duration of reproductive years after a series of exclusions in the GBCS. $: Postmenopausal women born before 1940 were analyzed; model 1: a crude hazard ratio model without adjustments; model 2: a multivariate adjust model including age, diabetes, hypertension, dyslipidemia, smoking, alcohol drinking, physical activity, body mass index, self-rated health, education, job, family income, number of children and oral contraceptive pill use. **Table 4.** Baseline characteristics of two birth cohorts in the GBCS. Hypertension: systolic blood pressure, ≥ 140 mmHg, diastolic blood pressure, ≤ 90 mmHg, medication or diagnosed; diabetes: fasting blood glucose ≥ 7 or medication or diagnosis; dyslipidaemia: total cholesterol ≥ 5.2 mmol/L, triglyceride ≥ 1.7 mmol/L, low density lipoprotein ≥ 3.4 mmol/L, high density lipoprotein < 1.0 mmol/L, medication or diagnosis; BMI: body mass index. SD: standard deviation. **Table 5.** Baseline characteristics of two types of samples in the GBCS. Hypertension: systolic blood pressure, ≥ 140 mmHg, diastolic blood pressure, ≤ 90 mmHg, medication or diagnosed; diabetes: fasting blood glucose ≥ 7 or medication or diagnosis; dyslipidaemia: total cholesterol ≥ 5.2 mmol/L, triglyceride ≥ 1.7 mmol/L, low density lipoprotein ≥ 3.4 mmol/L, high density lipoprotein < 1.0 mmol/L, medication or diagnosis; BMI: body mass index. SD: standard deviation. Q25: the 25th quartile; Q75: the 75th quartile.

## Data Availability

Data used in this analysis are available from the corresponding author on reasonable request.

## Abbreviations

HR: Hazard ratio
CI: Confidence interval
GBCS: Guangzhou Biobank Cohort Study
GZCDC: Guangzhou Centers for Disease Control and Prevention
ICD: International Classification of Diseases
IVT: Intravenous thrombolysis
IPAQ: International Physical Activity Questionnaire
OC: Oral contraceptive
