# Supplementary material for: Age at menarche, age at menopause, reproductive years and risk of fatal stroke occurrence among Chinese women: the Guangzhou Biobank Cohort Study
Source: BMC Womens Health. 2021 Dec 28;21:433. doi: 10.1186/s12905-021-01579-9 (PMC8714414; doi:10.1186/s12905-021-01579-9)
Supplement: Supplementary file 1 — Additional file 1. Table 1. Association between fatal stroke occurrence and age at menarche after a series of exclusions in the GBCS$: Postmenopausal women born before 1940 were analyzed; model 1: a crude hazard ratio model without adjustments; model 2: a multivariate adjust model including age, diabetes, hypertension, dyslipidemia, smoking, alcohol drinking, physical activity, body mass index, self-rated health, education, job, family income, number of children and oral contraceptive pill use. Table 2. Association between fatal stroke occurrence and age at menopause after a series of exclusions in the GBCS. $: Postmenopausal women born before 1940 were analyzed; model 1: a crude hazard ratio model without adjustments; model 2: a multivariate adjust model including age, diabetes, hypertension, dyslipidemia, smoking, alcohol drinking, physical activity, body mass index, self-rated health, education, job, family income, number of children and oral contraceptive pill use. Table 3. Association between stroke mortality and duration of reproductive years after a series of exclusions in the GBCS. $: Postmenopausal women born before 1940 were analyzed; model 1: a crude hazard ratio model without adjustments; model 2: a multivariate adjust model including age, diabetes, hypertension, dyslipidemia, smoking, alcohol drinking, physical activity, body mass index, self-rated health, education, job, family income, number of children and oral contraceptive pill use. Table 4. Baseline characteristics of two birth cohorts in the GBCS. Hypertension: systolic blood pressure, ≥ 140 mmHg, diastolic blood pressure, ≤ 90 mmHg, medication or diagnosed; diabetes: fasting blood glucose ≥ 7 or medication or diagnosis; dyslipidaemia: total cholesterol ≥ 5.2 mmol/L, triglyceride ≥ 1.7 mmol/L, low density lipoprotein ≥ 3.4 mmol/L, high density lipoprotein < 1.0 mmol/L, medication or diagnosis; BMI: body mass index. SD: standard deviation. Table 5. Baseline characteristics of two types of samples [file 12905_2021_1579_MOESM1_ESM.docx]

**Supplementary table 1** Association between fatal stroke occurrence and age at menarche after a series of exclusions in the GBCS

|  | Age at menarche (years) | | | | | | | | | |
| --- | --- | --- | --- | --- | --- | --- | --- | --- | --- | --- |
|  | ≤12 | 13 | 14 | | 15 | | 16 | | 17 | ≥18 |
| **Total Cohort** | 1099 | 2209 | 2388 | | 2394 | | 2322 | | 1498 | 2175 |
| No. of deaths | 10 (0.010) | 25 (0.021) | 26 (0.011) | 22 (0.009) | | | | 34 (0.015) | 30 (0.02) | 46 (0.021) |
| Model 1 (HR, 95% CI) | 1.07(0.51-2.25),*P*=0.87 | 1.26 (0.71-2.23), *P*=0.44 | 1.23 (0.70-2.16), *P*=0.48 | | 1.00 | | 1.59 (0.93-2.72), *P*=0.09 | | 2.18 (1.26-3.79), *P*=0.005 | 2.32 (1.40-3.86), *P*=0.001 |
| Model 2 (HR, 95% CI) | 1.56(0.73-3.31), *P*=0.25 | 1.75 (0.98-3.13), *P*=0.06 | 1.51 (0.86-2.68), *P*=0.16 | | 1.00 | | 1.38 (0.80-2.36), *P*=0.25 | | 1.70 (0.97-2.95), *P*=0.06 | 1.69 (1.01-2.82), *P*=0.04 |
| **Born < 1940^$^ Cohort** | 227 | 453 | 591 | | 807 | | 814 | | 614 | 944 |
| No. of deaths | 10 (0.044) | 14 (0.031) | 19 (0.032) | 12 (0.015) | | 22 (0.027) | | | 24 (0.039) | 42 (0.044) |
| Model 1(HR, 95% CI) | 3.01 (1.30-6.98), *P*=0.01 | 2.06 (0.95-4.45), *P*=0.07 | 2.17 (1.05-4.47), *P*=0.04 | | 1.00 | | 1.77 (0.88-3.58), *P*=0.11 | | 2.61 (1.31-5.23), *P*=0.007 | 3.11 (1.64-5.91), *P*=0.001 |
| Model 2(HR, 95% CI) | 3.67 (1.56-8.60), *P*=0.003 | 2.23 (1.02-4.85), *P*=0.04 | 2.37 (1.14-4.88), *P*=0.02 | | 1.00 | | 1.57 (0.77-3.18), *P*=0.21 | | 2.30 (1.14-4.62), *P*=0.02 | 2.56 (1.34-4.89), *P*=0.004 |
| **Born ≥1940^&^ Cohort** | 872 | 1756 | 1797 | | 1587 | | 1508 | | 884 | 1231 |
| No. of deaths | - | 11 (0.006) | 7 (0.004) | | 1.00 | | 12 (0.008) | | 6 (0.007) | 4 (0.003) |
| Model 1 (HR, 95% CI) | - | 1.01 (0.43-2.38), *P*=0.98 | 0.64 (0.25-1.69), *P*=0.37 | | 1.00 | | 1.28 (0.55-2.96), *P*=0.57 | | 1.09 (0.40-2.99), *P*=0.87 | 0.51 (0.16-1.64), *P*=0.26 |
| Model 2 (HR, 95% CI) | - | 1.14 (0.48-2.71), *P*=0.77 | 0.66 (0.25-1.75), *P*=0.40 | | 1.00 | | 1.25 (0.54-2.90), *P*=0.61 | | 0.98 (0.35-2.72), *P*=0.97 | 0.47 (0.14-1.50), *P*=0.20 |

$: Postmenopausal women born before 1940 were analyzed; model 1: a crude hazard ratio model without adjustments; model 2: a multivariate adjust model including age, diabetes, hypertension, dyslipidemia, smoking, alcohol drinking, physical activity, body mass index, self-rated health, education, job, family income, number of children and oral contraceptive pill use.

**Supplementary table 2** Association between fatal stroke occurrence and age at menopause after a series of exclusions in the GBCS

|  | Age at menopause (years) | | | | | | | | |
| --- | --- | --- | --- | --- | --- | --- | --- | --- | --- |
|  | <43 | 43-47 | 48-50 | | | 51-52 | ≥53 | | |
| Total cohort | 662 | 2201 | 5722 | | | 2609 | 2891 | | |
| No. of deaths | 23 (0.035) | 33 (0.015) | 77 (0.013) | 28 (0.011) | | | | | 32 (0.011) |
| Model 1 (HR, 95% CI) | 3.17 (1.83-5.51),*P*<0.001 | 1.38 (0.83-2.29),*P*=0.21 | 1.24 (0.80-1.91), *P*=0.33 | | | 1.00 | 0.99 (0.60-1.65), *P*=0.97 | | |
| Model 2 (HR, 95% CI) | 1.79 (1.03-3.14), P=0.04 | 1.02 (0.61-1.69), P=0.95 | 1.08 (0.70-1.67), P=0.73 | | | 1.00 | 1.02 (0.61-1.69), P=0.95 | | |
| Born < 1940^$^ Cohort | 348 | 864 | 1816 | | | 698 | 724 | | |
| No. of deaths | 19 (0.055) | 29 (0.034) | 55 (0.030) | | 16 (0.023) | | | | 24 (0.033) |
| Model 1 (HR, 95% CI) | 2.49 (1.28-4.84),*P*=0.007 | 1.45 (0.79-2.67),*P*=0.23 | 1.31 (0.75-2.29), *P*=0.34 | | | 1.00 | 1.40 (0.75-2.64), *P*=0.29 | | |
| Model 2 (HR, 95% CI) | 2.07 (1.06-4.06), *P*=0.03 | 1.37 (0.74-2.54),*P*=0.31 | 1.28 (0.73-2.24), *P*=0.39 | | | 1.00 | 1.46 (0.77-2.75), *P*=0.24 | | |
| Born ≥1940^&^ Cohort | 314 | 1337 | 3906 | | | 1911 | 2167 | | |
| No. of deaths | 4 (0.013) | 4 (0.003) | 22 (0.006) | | 12 (0.006) | | | 8 (0.004) | |
| Model 1 (HR, 95% CI) | 1.90 (0.61-5.90),P=0.27 | 0.48 (0.15-1.49),*P*=0.20 | 0.89 (0.44-1.79), *P*=0.74 | | | 1.00 | 0.56 (0.23-1.37), *P*=0.21 | | |
| Model 2 (HR, 95% CI) | 1.29 (0.41-4.07), *P*=0.66 | 0.42 (0.14-1.32),*P*=0.14 | 0.84 (0.41-1.70), *P*=0.63 | | | 1.00 | 0.50 (0.20-1.23), *P*=0.50 | | |

$: Postmenopausal women born before 1940 were analyzed; model 1: a crude hazard ratio model without adjustments; model 2: a multivariate adjust model including age, diabetes, hypertension, dyslipidemia, smoking, alcohol drinking, physical activity, body mass index, self-rated health, education, job, family income, number of children and oral contraceptive pill use.

**Supplementary table 3** Association between stroke mortality and duration of reproductive years after a series of exclusions in the GBCS

|  | Duration of reproductive years | | | | |
| --- | --- | --- | --- | --- | --- |
|  | ≤28 | 29-31 | 32-34 | 35-37 | ≥38 |
| *Stroke* | | | | | |
| **Total cohort** | 1151 | 1815 | 3388 | 4482 | 3249 |
| No. of deaths | 39 (0.034) | 28 (0.015) | 42 (0.012) | 52 (0.012) | 32 (0.010) |
| Model 1 (HR, 95% CI) | 2.75 (1.78-4.26), *P*<0.001 | 1.23 (0.76-1.98), *P*=0.40 | 1.00 | 0.94 (0.62-1.41), *P*=0.76 | 0.80 (0.50-1.26), *P*=0.33 |
| Model 2 (HR, 95% CI) | 1.84 (1.18-2.86), *P*=0.007 | 1.01 (0.63-1.64), *P*=0.96 | 1.00 | 1.16 (0.77-1.75), *P*=0.47 | 1.13 (0.71-1.80), *P*=0.62 |
| **Born < 1940^$^ cohort** | 620 | 781 | 1160 | 1186 | 703 |
| No. of deaths | 33 (0.053) | 26 (0.033) | 31 (0.027) | 31 (0.026) | 22 (0.031) |
| Model 1 (HR, 95% CI) | 2.08 (1.27-3.39), *P*=0.003 | 1.26 (0.75-2.13), *P*=0.38 | 1.00 | 0.98 (0.59-1.61), *P*=0.93 | 1.16 (0.67-2.00), *P*=0.59 |
| Model 2 (HR, 95% CI) | 1.78 (1.08-2.92), *P*=0.02 | 1.18 (0.70-2.00), *P*=0.53 | 1.00 | 1.01 (0.61-1.67), *P*=0.97 | 1.34 (0.77-2.34), *P*=0.30 |
| **Born ≥1940^&^ cohort** | 531 | 1034 | 2228 | 3296 | 2546 |
| No. of deaths | 6 (0.011) | 2 (0.002) | 11 (0.005) | 21(0.003) | 10 (0.004) |
| Model 1 (HR, 95% CI) | 2.27 (0.84-6.13), *P*=0.11 | 0.38 (0.09-1.73), *P*=0.21 | 1.00 | 1.28 (0.62-2.66), *P*=0.50 | 0.78 (0.33-1.85), *P*=0.58 |
| Model 2 (HR, 95% CI) | 1.74 (0.64-4.74), *P*=0.28 | 0.35 (0.08-1.57), *P*=0.17 | 1.00 | 1.39 (0.67-2.90), *P*=0.38 | 0.80 (0.34-1.89), *P*=0.60 |

$: Postmenopausal women born before 1940 were analyzed; model 1: a crude hazard ratio model without adjustments; model 2: a multivariate adjust model including age, diabetes, hypertension, dyslipidemia, smoking, alcohol drinking, physical activity, body mass index, self-rated health, education, job, family income, number of children and oral contraceptive pill use.

**Supplementary Table 4** Baseline characteristics of two birth cohorts in the GBCS

| Characteristics | born <1940 | Born≥1940 |
| --- | --- | --- |
| Number, n | 4938 | 11566 |
| Age (years), mean±SD | 69.9±3.7 | 57.8±4.0 |
| Follow-up duration (years), mean±SD | 11.7±2.8 | 11.8±1.7 |
| Hypertension, % | 34.8 | 23.7 |
| Diabetes, % | 17.8 | 11.2 |
| Dyslipidaemia, % | 85.1 | 85.9 |
| BMI (kg/m2), mean±SD | 23.7±3.4 | 23.9±3.3 |
| Good/very good self-rated health, % | 81.8 | 83.1 |
| Oral contraceptive pill use, % | 12.5 | 20.0 |
| Current smoker, % | 3.8 | 1.1 |
| Current drinking, % | 14.5 | 23.2 |
| Active physical activity, % | 52.3 | 52.7 |
| Education ≤ primary, % | 71.9 | 40.6 |
| Manual job, % | 63.6 | 50.6 |
| Family income < 30000 CNY/year, % | 47.7 | 34.3 |
| Number of children born > 3, % | 46.2 | 10.8 |
| Age at menarche (years), mean±SD | 15.7±2.1 | 14.9±2.0 |
| Age at menopause (years), mean±SD | 48.9±4.1 | 49.7±3.6 |
| Reproductive years (years), mean±SD | 33.2±4.6 | 34.8±4.0 |

### Hypertension: systolic blood pressure, ≥140 mmHg, diastolic blood pressure, ≤90 mmHg, medication or diagnosed; diabetes: fasting blood glucose ≥7 or medication or diagnosis; dyslipidaemia: total cholesterol ≥ 5.2 mmol/L, triglyceride ≥1.7 mmol/L, low density lipoprotein ≥3.4 mmol/L, high density lipoprotein <1.0 mmol/L, medication or diagnosis; BMI: body mass index. SD: [standard deviation](http://www.baidu.com/link?url=bndwMnILK9f5jzcw3ZkWRwliCqGgjTxnODSdwzsqT1gEOFYDHJ64x8J_njQhpN6IHceqa1SdNiOqCU9UtTFXjrEhAWE_q6G8X4VpIlAI37XiEGwN-aRtHkGzhfUJFL7K).

**Supplementary Table 5** Baseline characteristics of two types of samples in the GBCS

| Characteristics | Study sample | Excluded sample |
| --- | --- | --- |
| Number, n | 16504 | 3839 |
| Age (years), mean±SD | 61.4±6.8 | 59.9±7.8 |
| Follow-up duration (years), mean±SD | 11.7±2.1 | 11.4±2.1 |
| Hypertension, % | 27.0 | 34.1 |
| Diabetes, % | 13.1 | 13.8 |
| Dyslipidaemia, % | 85.7 | 85.1 |
| BMI (kg/m2), mean±SD | 23.8 | 24.2±3.4 |
| Good/very good self-rated health, % | 82.7 | 75.8 |
| Oral contraceptive pill use, % | 17.8 | 17.1 |
| Current smoker, % | 1.9 | 1.7 |
| Current drinking, % | 20.6 | 24.4 |
| Active physical activity, % | 52.6 | 51.6 |
| Education ≤ primary, % | 49.9 | 39.7 |
| Manual job, % | 54.5 | 45.2 |
| Family income < 30000 CNY/year, % | 38.3 | 35.7 |
| Number of children born > 3, % | 21.4 | 16.2 |
| Age at menarche (years), median(Q25,Q75) | 15(14,17) | 15(13,16) |
| Age at menopause (years), median(Q25,Q75) | 50(48,52) | 50(46,52) |
| Reproductive years (years), median(Q25,Q75) | 35(32,37) | 35(31,37) |

Hypertension: systolic blood pressure, ≥140 mmHg, diastolic blood pressure, ≤90 mmHg, medication or diagnosed; diabetes: fasting blood glucose ≥7 or medication or diagnosis; dyslipidaemia: total cholesterol ≥ 5.2 mmol/L, triglyceride ≥1.7 mmol/L, low density lipoprotein ≥3.4 mmol/L, high density lipoprotein <1.0 mmol/L, medication or diagnosis; BMI: body mass index. SD: [standard deviation](http://www.baidu.com/link?url=bndwMnILK9f5jzcw3ZkWRwliCqGgjTxnODSdwzsqT1gEOFYDHJ64x8J_njQhpN6IHceqa1SdNiOqCU9UtTFXjrEhAWE_q6G8X4VpIlAI37XiEGwN-aRtHkGzhfUJFL7K). Q25: the 25^th^ quantile; Q75: the 75^th^ quantile.
